# Characterization of the complete mitochondrial genomes of *Maiestas dorsalis* and *Japananus hyalinus* (Hemiptera: Cicadellidae) and comparison with other Membracoidea

**DOI:** 10.1038/s41598-017-14703-3

**Published:** 2017-10-27

**Authors:** Yimin Du, Chunni Zhang, Christopher H. Dietrich, Yalin Zhang, Wu Dai

**Affiliations:** 1 Key Laboratory of Plant Protection Resources and Pest Management of Ministry of Education, College of Plant Protection, Northwest A&F University, Yangling, Shaanxi China; 2 Illinois Natural History Survey, Prairie Research Institute, University of Illinois at Urbana-Champaign, Champaign, Illinois United States of America

## Abstract

Only six mitochondrial genomes (mitogenomes) have been previously published for Cicadellidae, the largest family of Hemiptera. This study provides complete, annotated mitogenomes of two additional cicadellid, species *Maiestas dorsalis* and *Japananus hyalinus*, and the first comparative mitogenome analysis across the superfamily Membracoidea. The mitogenomes of both sequenced species are similar to those of other studied hemipteran mitogenomes in organization and the lengths are 15,352 and 15,364 bp with an A + T content of 78.7% and 76.6%, respectively. In *M. dorsalis*, all sequenced genes are arranged in the putative ancestral insect gene arrangement, while the tRNA cluster *trnW-trnC-trnY* is rearranged to *trnY-trnW-trnC* in *J. hyalinus*, the first reported gene rearrangement in Membracoidea. Phylogenetic analyses of the 11 available membracoid mitogenomes and outgroups representing the other two cicadomorphan superfamilies supported the monophyly of Membracoidea, and indicated that treehoppers are a derived lineage of leafhoppers. ML and BI analyses yielded topologies that were congruent except for relationships among included representatives of subfamily Deltocephalinae. Exclusion of third codon positions of PCGs improved some node support values in ML analyses.

## Introduction

Insect mitochondrial genomes (mitogenomes) are typically double-stranded circular molecules with 37 genes, including 13 protein-coding genes (PCGs), two ribosomal RNAs (rRNAs), and 22 transfer RNAs (tRNAs)^1^. Complete mitochondrial genome sequences are not only more phylogenetically informative than shorter sequences of individual genes, but also provide sets of genome-level characters, such as the relative positions of different genes, RNA secondary structures and modes of control of replication and transcription. Because of the abundance of mitochondria in cells, maternal inheritance, absence of introns, and high evolutionary rates, insect mitochondrial genome sequences are the most extensively used genomic marker(s) in insects and are becoming increasingly important for studies of insect molecular evolution, phylogeny and phylogeography^2–4^. Following the development of next generation sequencing technology, large numbers of mitochondrial genomes are becoming available^2,5^. Mitochondrial genomes representing each of the major subordinal lineages from each of the 28 recognized insect orders are now available and representation at the family level is steadily improving.

The hemipterous superfamily Membracoidea (leafhoppers and treehoppers) is of interest because it is the most diverse and successful lineage of sap-sucking phytophagous insects, and because of the great variety of behavioral and life-history strategies found within this group.

The phylogenetic relationships of the leafhopper family Cicadellidae to the treehopper families Membracidae, Melizoderidae, and Aetalionidae, all comprising superfamily Membracoidea, have been controversial for decades and conflicts among the few morphology-based phylogenetic hypotheses proposed by different authors for Membracoidea have not yet been resolved. Although, traditionally, Cicadellidae has been regarded as the sister group of a lineage comprising the three treehopper families mainly supported by morphological characters of the adults^6–8^, studies based on morphological^9,10^, paleontological^11^ and behavioral^12^ evidence suggest that the Cicadellidae are paraphyletic with respect to Membracidae. Molecular phylogenetic analysis of deep-level membracoid relationships based on 28S rRNA sequence data also indicated that Cicadellidae is paraphyletic with respect to Membracidae^13^. Within the Cicadellidae, the relationships among family-group taxa based on recent phylogenetic analyses of DNA sequence and morphological data remain poorly resolved and no family-group classification has yet gained universal acceptance^9,13–16^.

Until now, most studies of complete mitochondrial genomes in Hemiptera have focused on Heteroptera. To date, only nine complete mitochondrial genomes of Membracoidea have been deposited in GenBank, including the aetalionid *Darthula hardwickii* (KP316404)^17^, the membracids *Leptobelus gazella* (JF801955)^18^ and *Entylia carinata* (KX495488)^19^, and the cicadellids *Drabescoides nuchalis* (KR349344)^20^, *Nephotettix cincticeps* (KP749836), *Tambocerus sp*.(KT827824)^21^, *Empoasca vitis* (KJ815009)^22^ (Qin *et al*.^23^ recently showed that Chinese specimens previously identified as *E. vitis* are actually *E. onukii* so the specimens used by Zhou *et al*.^22^ were probably misidentified because true *E. vitis* is a European species not known to occur in China), *Homalodisca vitripennis* (AY875213; listed as “*H. coagulata*”) and *Idioscopus nitidulus* (KR024406). So far, the secondary structures of tRNAs and rRNAs of these species have not been predicted and the nucleotide sequence data have not been used to estimate phylogenetic relationships. The number of sequenced cicadellid mitochondrial genomes also remains very limited relative to the species-richness of Cicadellidae. Because mitochondrial genomes are available for only three species of Deltocephalinae, the largest cicadellid subfamily, representing only three of the 39 recognized tribes, more taxa and more data are needed for future phylogenetic studies to obtain a more resolved and supported phylogeny of the subfamily.

Here we present and analyze the complete mitochondrial genomes of two additional deltocephaline species *Maiestas dorsalis* (Motschulsky) (tribe Deltocephalini) and *Japananus hyalinus* (Osborn) (tribe Opsiini), including the gene order, nucleotide composition, codon usage, tRNA secondary structure, rRNA secondary structure, gene overlaps and non-coding regions. Using these new sequences, along with those of previously published mitochondrial genomes of Membracoidea, we reconstructed the phylogenetic relationships among them based on the concatenated nucleotide sequences of 13 protein-coding genes (PCGs) and two ribosomal RNA genes.

## Materials and Methods

### Sample collection and DNA extraction

Adults of *M. dorsalis* used in this study were collected in Guilin, Guangxi province, China (25°63′N,109°91′E, July 2015), while *Japananus hyalinus* specimens were collected in Yangling, Shaanxi province, China (34°27′N, 108°09′E, September 2014). Fresh specimens were initially preserved in 100% ethanol, and then stored at −20 °C in the laboratory. After morphological identification, voucher specimens with male genitalia prepared were deposited in the Entomological Museum of Northwest A&F University, and total genomic DNA was extracted from muscle tissues of the thorax using the DNeasy DNA Extraction kit (Qiagen).

### Next generation sequence assembly

Most of the mitochondrial genome sequences of the two species were generated using Illumina HiSeq™2500 with paired reads of 2 × 150 bp. A total of 26,435,488 and 13,989,788 raw paired reads were retrieved and quality-trimmed using CLC Genomics Workbench v7.0.4 (CLC Bio, Aarhus, Denmark) with default parameters for *M. dorsalis* and *J. hyalinus* respectively. Subsequently, with the mitochondrial genome of *D. nuchalis* (KR349344)^20^ employed as reference, the resultant 26,435,023 and 13,989,519 clean paired reads were used for mitochondrial genome reconstruction using MITObim v1.7 software^24^ with default parameters. A total of 18,137 individual mitochondrial reads yielded an average coverage of 158.6 × for *M. dorsalis*, while for *J. hyalinus* a total of 17,924 individual mitochondrial reads yielded an average coverage of 70.3 × .

### Gap closing-PCR amplification and sequencing

According to the flanking sequences assembled from the NGS data, we designed two pairs of primers to amplify the control region (CR) (*M. dorsalis*: Forward 5′-3′: TGTATAACCGCGAATGCTGGCACAA and Reverse 5′-3′: TTAGGGTATGAACCTAATAGCT; *J. hyalinus*: Forward 5′-3′: ATAGCCAGAATCAAACCT and Reverse 5′-3′: AAGTGTCACAGGCTTAGGT). PCR reactions were performed with TaKaRa LA-Taq Kits (TaKaRa Co., Dalian, China) under the following cycling conditions: 5 min at 94 °C, 38 cycles of 30 s at 94 °C, 1 min at 45–50 °C, 3 min at 68 °C, and a final elongation step at 68 °C for 15 min. PCR products were eletrophoresed on 1% agarose gels, purified and then sequenced in both directions on an ABI 3730 XL automated sequencer (Applied Biosystems).

### Genome annotation and bioinformatic analyses

The two mitochondrial genomes were annotated with GENEIOUS R8 (Biomatters Ltd., Auckland, New Zealand). All 13 protein-coding genes and 2 rRNA genes were determined by comparison with the homologous sequences of other leafhoppers from GenBank. The 22 tRNA genes were identified using both of the tRNAScan-SE server v 1.21^25^ and MITOS WebSever^26^, the secondary structure was also predicted by the MITOS WebSever.

The base composition and relative synonymous codon usage (RSCU) values of each protein coding gene (PCG) were calculated with MEGA 6.06^27^. Strand asymmetry was calculated using the formulas AT skew = [A−T]/[A + T] and GC skew = [G−C]/[G + C]^28^. The number of nonsynonymous substitutions per nonsynonymous site (Ka) for the two species were calculated with DnaSP 5.0^29^, using *Magicicada tredecim* (Riley) from Cicadoidea and *Callitettix braconoides* (Walker)^30^ from Cercopoidea as references. The tandem repeats of the A + T-rich region were identified by the tandem repeats finder online server (http://tandem.bu.edu/trf/trf.html)^31^.

### Phylogenetic analyses

#### Taxa selection

Besides the two mitochondrial genomes obtained in this study, another nine available mitochondrial genomes for Membracoidea were downloaded from NCBI for phylogenetic analyses. A cicada, *Magicicada tredecim*, and a cercopid, *Callitettix braconoides*, were selected as outgroups. The ingroup species represented three families, Membracidae, Aetalionidae and Cicadellidae (Table 1).

**Table 1:** Taxonomic information and GenBank accession numbers for the species used in this study.

Superfamily	Family	Subfamily	Tribe	Species	Accession number	Reference
Cicadoidea	Cicadidae	Cicadettinae	Taphurini	*Magicicada tredecim*	KM000130	Direct Submission
Cercopoidea	Cercopidae	Callitettixinae	Callitettixini	*Callitettix braconoides*	JX844628	Liu *et al*., 2014^30^
Membracoidea	Membracidae	Centrotinae	Leptobelini	*Leptobelus gazella*	JF801955	Zhao and Liang, 2016^18^
		Smiliinae	Polyglyptini	*Entylia carinata*	KX495488	Mao *et al*., 2016^19^
	Aetalionidae	Darthulinae	Darthulini	*Darthula hardwickii*	KP316404	Liang *et al*., 2016^17^
	Cicadellidae	Cicadellinae	Proconiini	*Homalodisca vitripennis*	AY875213	Direct Submission
		Typhlocybinae	Empoascini	*Empoasca vitis*	KJ815009	Zhou *et al*., 2016^22^
		Eurymelinae	Idiocerini	*Idioscopus nitidulus*	KR024406	Direct Submission
		Deltocephalinae	Chiasmini	*Nephotettix cincticeps*	KP749836	Direct Submission
			Drabescini	*Drabescoides nuchalis*	KR349344	Wu *et al*., 2016^20^
			Athysanini	*Tambocerus* sp.	KT827824	Yu *et al*., 2015^21^
			Deltocephalini	*Maiestas dorsalis*	KX786285	This study
			Opsiini	*Japananus hyalinus*	KY129954	This study

#### Sequence alignment and substitution saturation test

Sequences of all 13 PCGs and two rRNA genes were used in our analyses. After excluding stop codons, each PCG was aligned individually with codon-based multiple alignments using the MAFFT algorithm in the TranslatorX online server (http://translatorx.co.uk/)^32^, with gaps and ambiguous sites removed from the protein alignment before back-translating to nucleotides using GBlocks under default settings. The sequences of two rRNA genes were aligned separately using the MUSCLE algorithm implemented in MEGA 6.06. We found that MUSCLE and MAFFT have been shown to perform similarly on our rDNA sequence data but because MUSCLE is implemented in MEGA 6.06, which facilitates manual removal of poorly aligned positions, we used MUSCLE for these genes.

Saturation tests for different codon positions of PCGs and the two rRNA genes were performed, with the uncorrected p-distances plotted against the GTR distances. All distances were generated using PAUP⁄4.0 b10^33^. The slope, correlation coefficient and average GTR distance were used as measures of substitution saturation; the lower the slope, the greater the level of saturation^34–36^.

#### Dataset concatenation, partitioning and substitution model selection

Alignments of all genes were concatenated using SequenceMatrix 1.7.8^37^. Five datasets were generated: 1) P123: 13 PCGs with 9846 nucleotides; 2) P12: first and second codon positions of 13 PCGs with 6564 nucleotides; 3) P123R: 13 PCGs and two rRNAs with 11905 nucleotides; 4) P12R: first and second codon positions of 13 PCGs and two rRNAs with 8623 nucleotides; and 5) AA: amino acid sequences of 13 PCGs with 3282 amino acids.

Optimal nucleotide substitution models and partition strategies were chosen by PartitionFinder v1.1.1^38^. Under the “greedy” search algorithm, we chose “unlinked” to estimate branch lengths and used the Bayesian information criterion (BIC) as the metric for the partitioning scheme. Details of the best-fit schemes for ML and BI analysis are shown in Supplementary Table S3.

#### Phylogenetic inference

ML analyses were conducted using raxmlGUI 1.5^39^ under the GTRGAMMAI model, and the node reliability was assessed by performing 1000 rapid bootstrap replicates (BS). Bayesian analysis was performed using MrBayes 3.2.6^40^. Two simultaneous runs with eight independent chains were run for five million generations and trees were sampled every 1000 generations. After the average standard deviation of split frequencies fell below 0.01, the first 25% of the total samples were discarded as burn-in and the remaining trees were used to generate a consensus tree and calculate the posterior probabilities (PP).

## Results and Discussion

### General features of the two newly sequenced mitochondrial genomes

**Figure 1:**
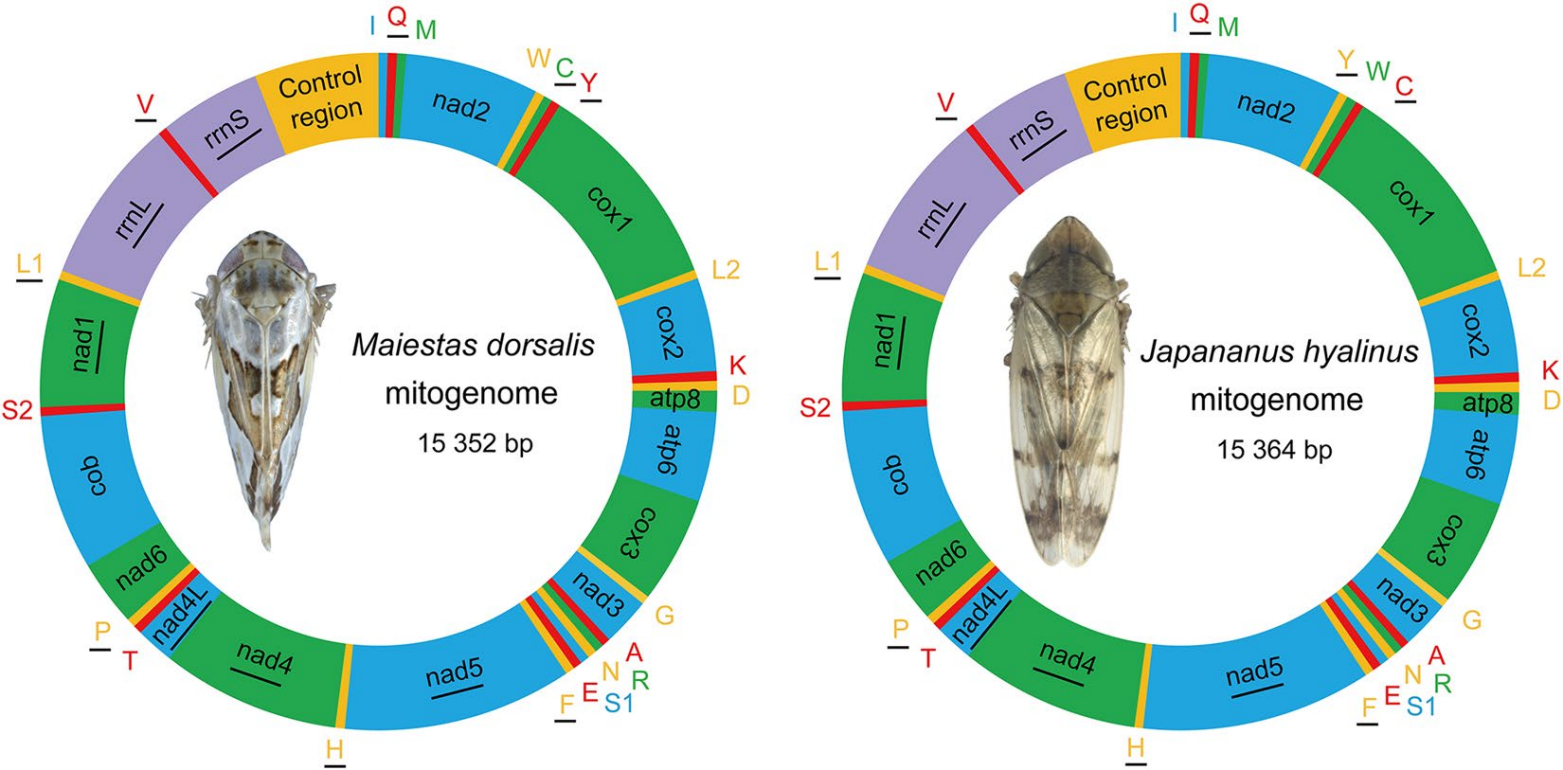
Circular map of the mitochondrial genome of *Maiestas dorsalis* and *Japananus hyalinus*. Protein coding and ribosomal genes are shown with standard abbreviations. Transfer RNA (tRNA) genes are indicated using the IUPAC-IUB single letter amino acid codes. Gene names without underlining indicate the direction of transcription in the major (J) strand, while names with underlining indicate transcription in the minor (N) strand.

The complete mitochondrial genomes of *M. dorsalis* (GenBank: KX786285) and *J. hyalinus* (GenBank: KY129954) are double-stranded circular molecules of length of 15352 bp and 15364 bp, respectively (Fig. 1). Both sizes were comparable to other sequenced Membracoidea mitochondrial genomes ranging from 14805 bp in *Nephotettix cincticeps* to 16007 bp in *Leptobelus gazella* (Table 2). Each mitochondrial genome included the 37 typical mitochondrial genes (13 PCGs, 22 tRNAs and two rRNAs) and a control region (A + T-rich region) (Supplementary Tables S1, S2).

**Table 2:** Nucleotide compositions in regions of the Membracoidea mitochondrial genomes.

Species	whole		PCGs		16S		12S		CR	
	length	AT%	length	AT%	length	AT%	length	AT%	length	AT%
*Leptobelus gazella*	16007	78.8	10920	77.0	1188	81.8	736	79.0	1750	88.4
*Entyliacarinata*	15662	78.1	10924	77.3	1171	82.0	722	78.3	1474	79.4
*Darthula hardwickii*	15355	78.0	10916	76.8	1198	82.3	737	78.0	1077	83.6
*Homalodisca vitripennis*	15304	78.4	10965	77.1	1201	80.9	728	77.9	1033	88.1
*Empoasca vitis*	15154	78.3	10947	76.7	1149	81.9	725	81.3	977	89.0
*Idioscopus nitidulus*	15287	78.7	10943	77.3	1196	80.3	734	78.6	991	89.1
*Nephotettix cincticeps*	14805	77.7	10901	76.9	1201	80.8	741	78.6	399	83.9
*Drabescoides nuchalis*	15309	75.6	10933	74.6	1197	79.1	740	78.9	956	77.3
*Tambocerus* sp.	15955	76.4	10963	74.3	1217	80.5	732	78.0	1581	86.0
*Maiestas dorsalis*	15352	78.8	10961	78.1	1217	81.4	745	79.7	908	81.5
*Japananus hyalinus*	15364	76.6	10954	75.8	1208	79.8	752	79.5	848	76.3

### Base composition

All the newly obtained mitochondrial genomes exhibited heavy AT nucleotide bias, with A + T% of the whole sequences 78.8% in *M. dorsalis* and 76.6% in *J. hyalinus*, similar to those found in other sequenced Membracoidea (75.6–78.8%)(Table 2). CR sequences in most species showed the strongest A + T% biases, except for *Entylia carinata*, *Drabescoides nuchalis* and *J. hyalinus*. All species had higher A + T% in *rrnL* than *rrnS*, and the PCGs showed the lowest A + T% among whole genes (Table 2). Except for *Empoasca vitis* and *J. hyalinus*, all species showed positive AT-skews (0.007–0.250), for both whole sequences and individual gene sequences. Except for the CR sequences in *L. gazella*, *E. carinata*, *Idioscopus nitidulus* and *J. hyalinus*, all other regions showed negative GC-skews (−0.073 to −0.345) (Supplementary Fig. S1).

### Gene rearrangement in Membracoidea

**Figure 2:**
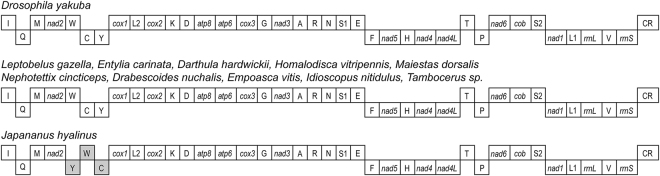
Linear comparison of mitochondrial genome organization in Membracoidea and *Drosophila yakuba*. The upper boxes indicate genes coding by J strand. Shaded boxes indicate genes involving mitochondrial genome arrangement.

Among the 11 sequenced mitochondrial genomes, the gene order of most species was highly conserved and identical to the putative ancestral insect (*Drosophila yakuba*) mitochondrial genome arrangement^1^. The only exception was *J. hyalinus*, the first known member of Membracoidea with a tRNA gene rearrangement, in which the tRNA cluster *trnW-trnC-trnY* was rearranged to *trnY-trnW-trnC* (Fig. 2). Although such gene rearrangements were previously unknown in the superfamily Membracoidea, they have often been found in other insect groups^41,42^.

### Protein-coding genes, codon usage and substitution rates

Of the 13 PCGs in *M. dorsalis* and *J. hyalinus*, nine were located on the majority strand (J-strand) while the other four PCGs were located on the minority strand (N-strand), as observed in other Membracoidea species (Supplementary Tables S1; S2). Among the concatenated 13 PCGs of each species of Membracoidea, the third codon position had an A + T content (84.3–91.7%) much higher than that of the first (70.1–74.7%) and second (67.8–70.0%) positions (Supplementary Fig. S2).

Most of the PCGs started with the standard ATN codon, except for *nad5*, which began with TTG, a pattern also observed in *Tambocerus* sp., *I. nitidulus* and *E. carinata*. Also, *atp8* in *H. vitripennis*, *D. hardwickii* and *I. nitidulus* started with TTG, and *cox2* in *Empoasca vitis* began with GTG. In *M. dorsalis*, *nad5* terminated with TAG, *cox2* and *nad1* ended with an incomplete T codon, with all other 10 PCGs using TAA as the T codon. In *J. hyalinus*, all PCGs terminated with TAA except for *cox2*, which ended with an incomplete T codon. Overall, in Membracoidea species, more TAA than TAG were used, and at least one incomplete T codon was present.

**Figure 3:**
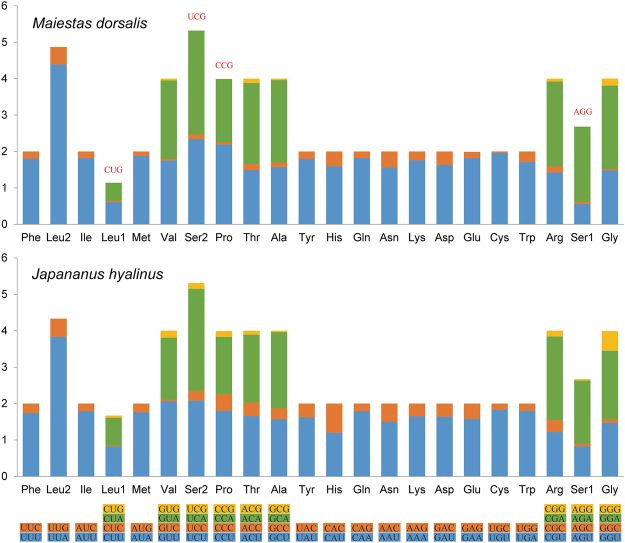
Relative synonymous codon usage (RSCU) of *Maiestas dorsalis* and *Japananus hyalinus* mitochondrial genomes. The stop codon is not given. Codons absent in mitochondrial genomes are shown at the top of columns.

For the relative synonymous codon usage (RSCU) of *M. dorsalis* and *J. hyalinus*, the four most frequently utilized amino acids were Leucine (Leu), Isoleucine (Ile), Methionine (Met) and Serine (Ser). Among the 62 available codons (excluding TAA and TAG), Leu (CUG), Ser (AGG, UCG) and Pro (CCG) were missing in *M. dorsalis* (Fig. 3).

**Figure 4:**
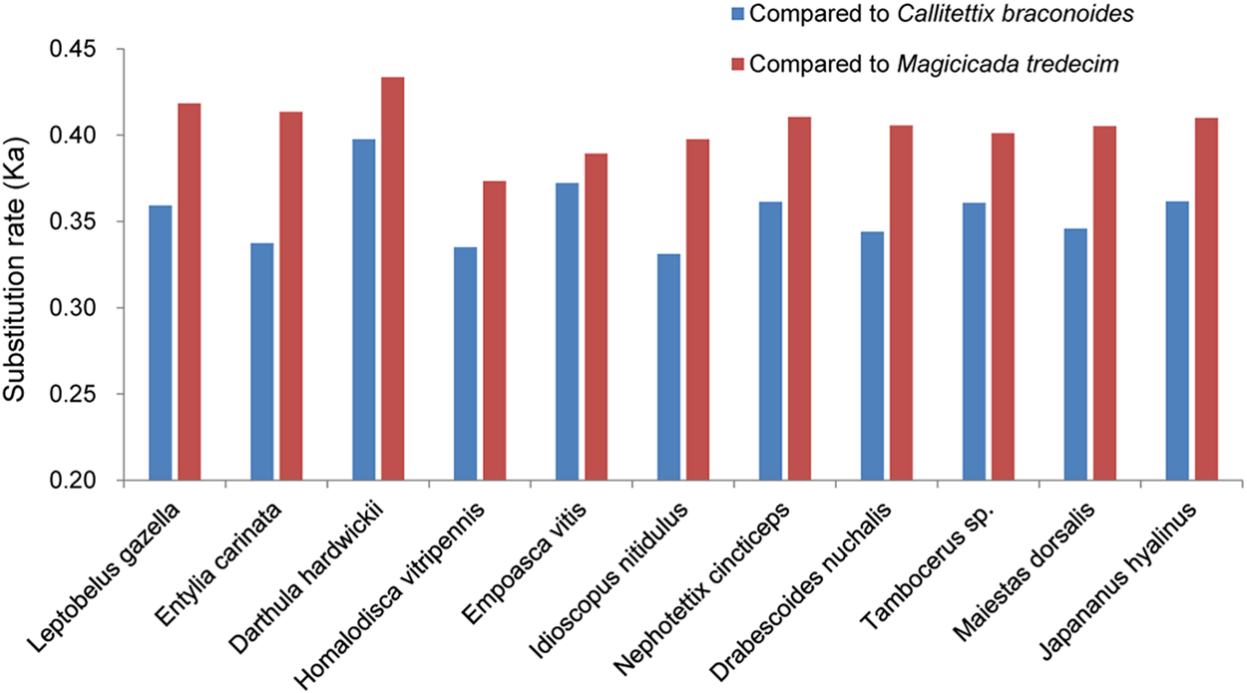
Comparison of substitution rates among Membracoidea mitochondrial genomes. The nonsynonymous substitution rate (Ka) was calculated in a pairwise fashion, using *Callitettix braconoides* and *Magicicada tredecim* as references.

The nonsynonymous substitution rate (Ka) for each taxon was measured in comparison with *Callitettix braconoides* and *Magicicada tredecim* (Fig. 4). Ka was relatively higher in treehoppers than leafhoppers when compared with *M. tredecim*, and the treehopper *D. hardwickii* (Aetalionidae) always showed the highest values, but Ka values were similar between these species (0.331–0.398 and 0.374–0.433) (Fig. 4).

### Transfer RNA and ribosomal RNA genes

All of the 22 typical animal tRNA genes were found in *M. dorsalis* and *J. hyalinus* mitochondrial genomes, and their anticodons are identical to those present in other Membracoidea (Supplementary Tables S1, S2). In both, 14 tRNAs were encoded by the J-strand and the remaining eight were encoded by the N-strand. The nucleotide length of these tRNA genes ranges from 63 bp to 72 bp in *M. dorsalis*, and from 61 bp to 73 bp in *J. hyalinus*. For the other nine membracoid species, *trnR* and *trnF* were not predicted in *H.vitripennis*.

Except for *trnS1* which lacks the dihydrouridine (DHU) stem and forms a simple loop, all tRNAs in the two new mitochondrial genomes could be folded into the typical cloverleaf secondary structure (Supplementary Figs S3, S4). Two extra single A nucleotides, 23 G-U mismatches, six U-U mismatches, two A-A mismatches and one C-U mismatch were found in *M. dorsalis* tRNA genes. While in *J. hyalinus* one extra single A nucleotide, 26 G-U mismatches, four U-U mismatches, one A-A mismatch, one A-C mismatch and one A-G mismatch were found.

Similar to other insect mitochondrial genomes, two *rrn* genes were encoded on the N-strand, and located between *trnL1* and *trnV*, and between *trnV* and the A + T-rich region, respectively. The lengths of the *rrnL* and *rrnS* in *M. dorsalis* were 1217 bp and 745 bp, with the A + T content 81.4% and 79.7%, respectively, while in *J. hyalinus* are 1208 bp and 752 bp, with the A + T content 79.8% and 79.5%, respectively, which were consistent with the data available for other Membracoidea species (Table 2).

### Gene overlaps and non-coding regions

The whole *M. dorsalis* mitochondrial genome had a total of 35 bp in overlaps between 11 gene junctions, while *J. hyalinus* had 27 bp overlaps between eight gene junctions. The longest overlap (8 bp) occurs between *trnW* and *trnC* in both two species. The two common pairs of gene overlaps: *atp8*-*atp6* (7 bp) and *nad4*-*nad4l* (7 bp), also were found in two other species (Supplementary Tables S1, S2).

There were 13 intergenic spacers totaling 92 bp non-coding bases in the *M. dorsalis* mitochondrial genome, ranging in size from 1 bp to 16 bp, and the longest two intergenic spacers (16 bp) were between *nad2-trnW* and *cox1-trnL2* respectively. For *J. hyalinus*, 15 intergenic spacers occupied 178 bp non-coding bases, the longest being 73 bp between *trnY* and *trnW*, caused by the tRNA gene rearrangement(Supplementary Tables S1, S2).

The putative control region, located between *rrnS* and *trnI*, was the longest intergenic spacer in the mitochondrial genome. The lengths of this region in *M. dorsalis* and *J. hyalinus* were 908 bp and 848 bp respectively, well within the range of other sequenced Membracoidea (399 bp in *N. cincticeps* to 1750 bp in *L. gazella*) (Table 1).

**Figure 5:**
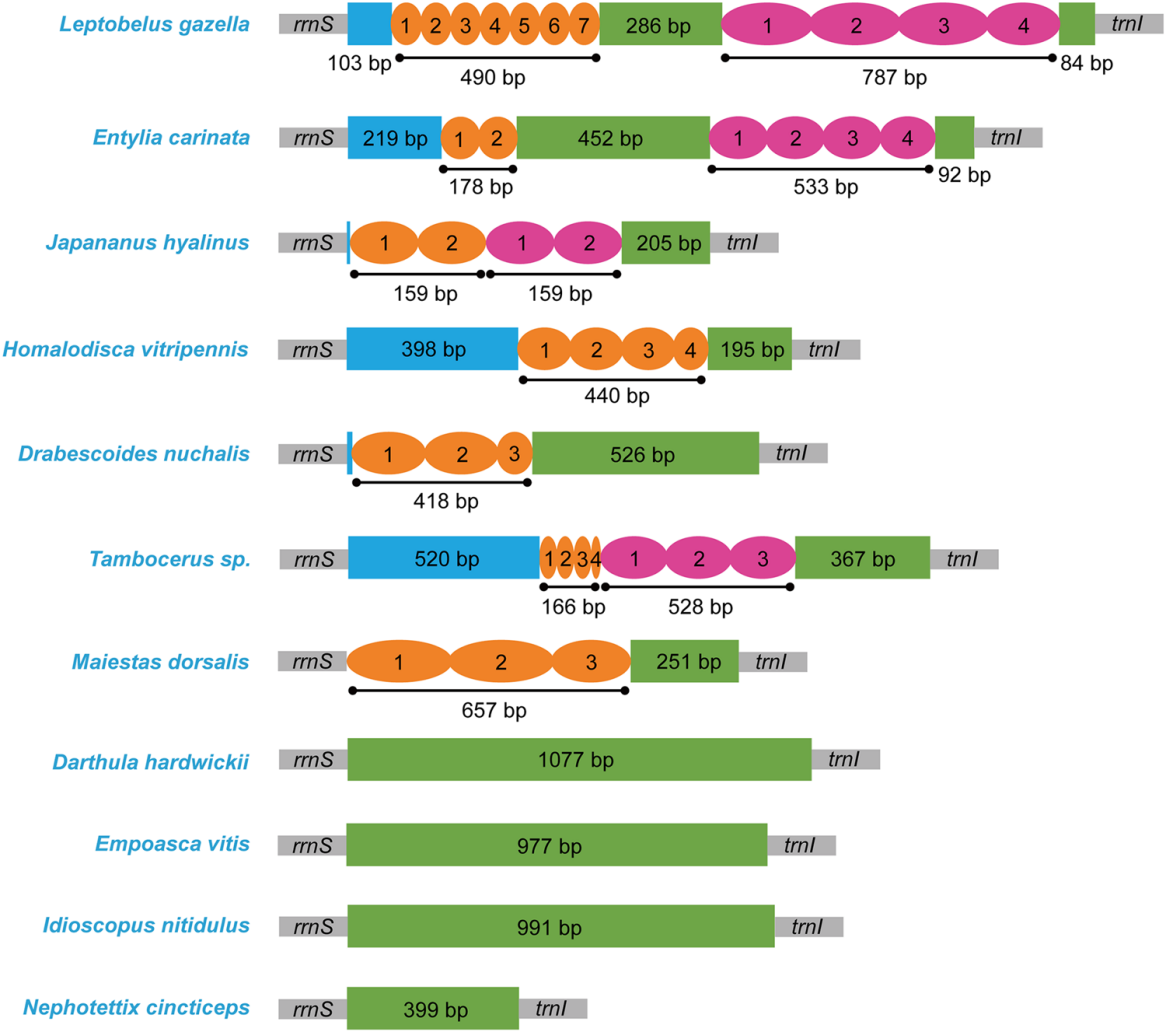
Organization of the control region in Membracoidea mitochondrial genomes. The colored ovals with Arabic numerals indicate the tandem repeats, the remaining regions are shown with colored boxes.

The control region sequence of *M. dorsalis* included one large tandem repeat, two 239 bp tandem repeat units and a partial third (179 bp) were beginning with the first nucleotide of this region (Fig. 5). *J. hyalinus* also had two different 159 bp tandem repeat units, consisting of the main portion of the control region. Tandem repeats identified in the control region of other sequenced Membracoidea mitochondrial genomes include four in *H. vitripennis*, three in *D. nuchalis*, and two different types of tandem repeats in *L. gazella*, *E. carinata* and *Tambocerus* sp. Characteristics of this region in Membracoidea were taxon-specific, and the different size or copy numbers of repeat units had some influence on the size of the region (Fig. 5).

### Phylogenetic relationships

**Figure 6:**
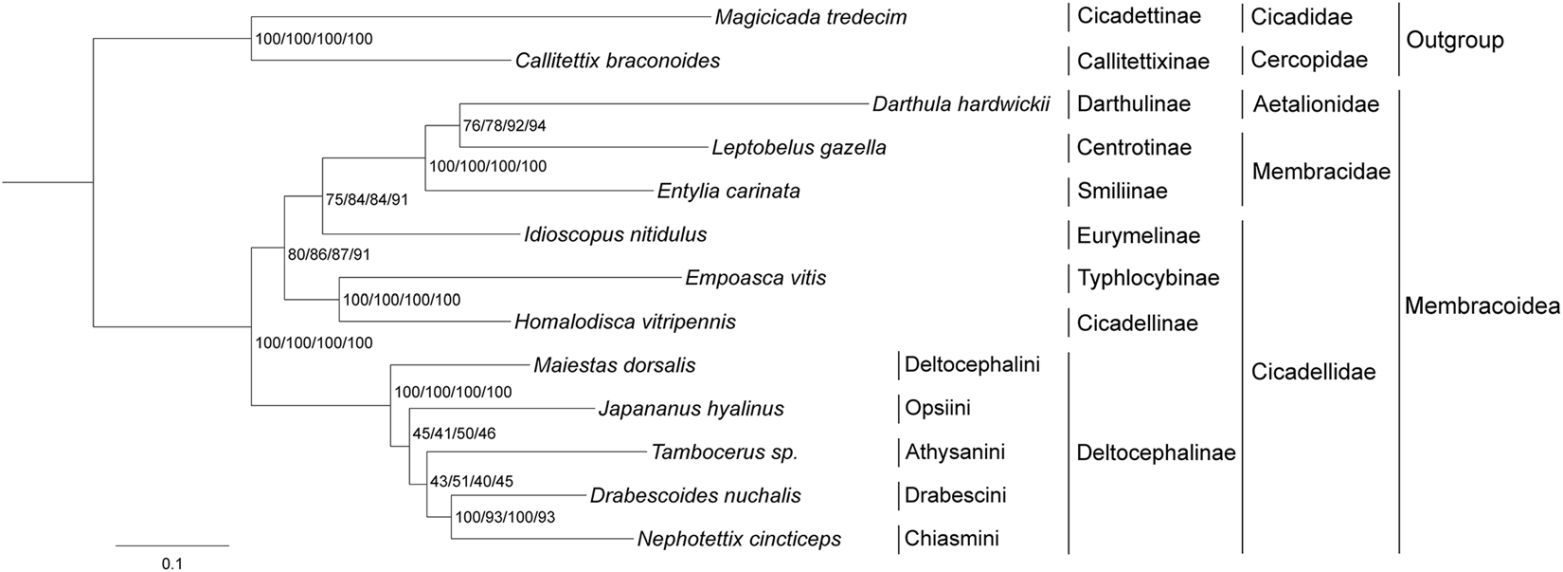
Membracoidea phylogeny based on the P123/P12/P123R/P12R datasets inferred from RAxML. Numbers on branches are bootstrap values (BS).

**Figure 7:**
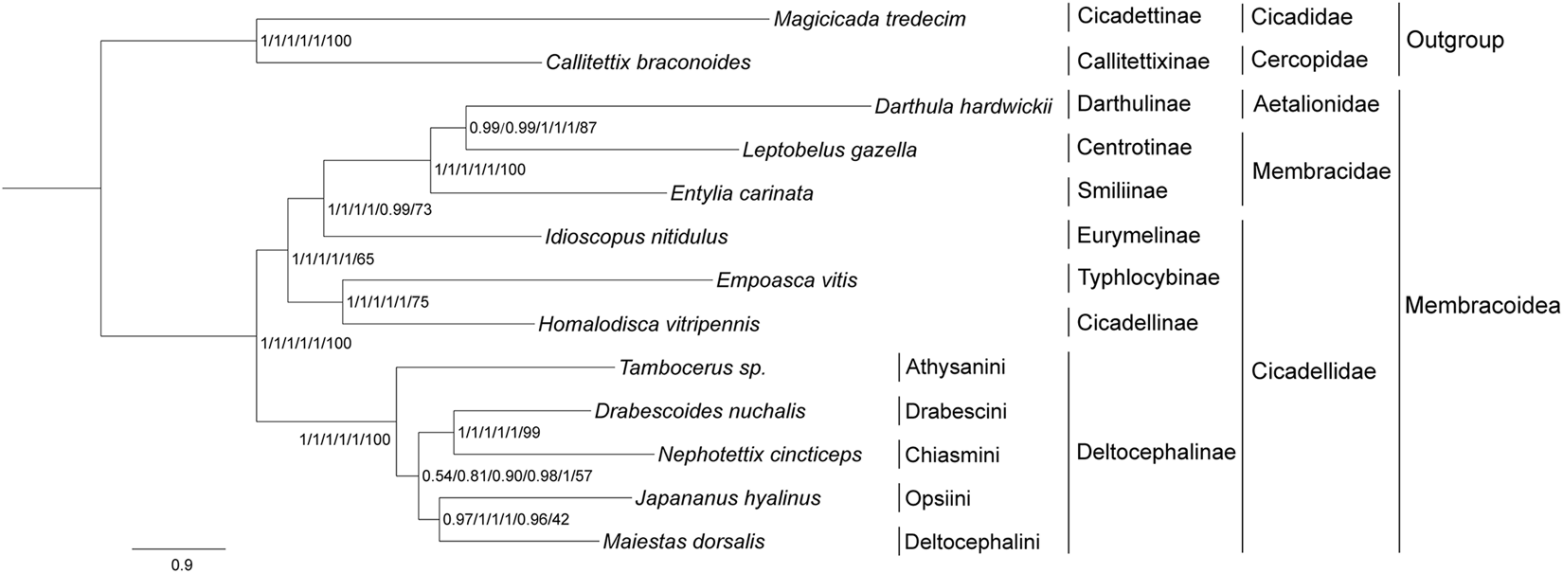
Membracoidea phylogeny based on the P123/P12/P123R/P12R/AA datasets obtained with MrBayes and AA dataset inferred from RAxML. Numbers on branches are Bayesian posterior probabilities (PP) and bootstrap values (BS).

We performed 10 independent phylogenetic analyses to evaluate the influence of different datasets and inference methods on tree topology and nodal support. These analyses yielded two different tree topologies, with incongruence restricted to relationships among members of cicadellid subfamily Deltocephalinae (Figs 6,7).

Monophyly at the superfamily level within Membracoidea was strongly supported in both trees (Figs 6,7). Derivation of Membracidae and Aetalionidae from within a paraphyletic Cicadellidae was well supported by all results, as suggested by previous analyses^9,13^. Although other recent analyses based on mitogenomic data supported treehoppers as a sister group to Cicadellidae^19–21^, none of these studies included all full available mitochondrial genomes within Membracoidea and, thus, the incongruence may be due to sample bias. For the three treehoppers, the two species of Membracidae *E. carinata* and *L. gazella* formed a paraphyletic grade giving rise to *D. hardwickii* (Aetalionidae), indicating paraphyly of Membracidae (Figs 6,7). This result differs from previous studies, in which Membracidae was usually placed as the sister clade to Aetalionidae^13,43,44^.

Within Cicadellidae, the eight species sampled in this study represent four subfamilies, Eurymelinae, Cicadellinae, Typhlocybinae and Deltocephalinae. The inferred relationships (Deltocephalinae + (Eurymelinae + (Cicadellinae + Typhlocybinae))) were incongruent with a previous phylogeny based on 28S sequences, in which the relationships (Cicadellinae + (Deltocephalinae + (Typhlocybinae + Eurymelinae))) were found^13^ (Figs 6,7). However, the latter topology received low branch support, which may explain the incongruence.

Within Deltocephalinae, the five species (*M. dorsalis*, *J. hyalinus*, *D. nuchalis*, *N. cincticeps* and *Tambocerus* sp.) representing five tribes (Deltocephalini, Opsiini, Drabescini, Chiasmini and Athysanini, respectively) formed a monophyletic group with high support. For all datasets in BI analyses and the AA dataset in ML analyses, the topology (Athysanini + ((Opsiini + Deltocephalini) + (Drabescini + Chiasmini))) was recovered, while except for the AA dataset, all other datasets yield the topology (Deltocephalini + (Opsiini + (Athysanini + (Drabescini + Chiasmini)))) (Figs 6, 7). Thus, relationships among the included Deltocephalinae were inconsistently resolved in the different analyses, with support for some branches relatively low (BS < 50, PP < 0.9). Thus, as in a previous phylogenetic study based on combined morphological, 28S and Histone H3 data^14^ our analyses were unable to resolve some relationships with confidence. Apparent topological discordances between our results and those of Zahniser and Dietrich^14^ may due to the small taxon sample of the current study. Classification of Deltocephalinae has been unstable, with *D. nuchalis* formerly placed in a separate subfamily, Selenocephalinae^45,46^, but more recently placed into Deltocephalinae based on morphological characters^47,48^. Recent phylogenetic studies indicate that the subfamily as defined by Oman *et al*.^49^ was not monophyletic and that several other leafhopper subfamilies defined by Oman *et al*.^49^ had their closest relatives within the Deltocephaline lineage^13,14,50^.

**Figure 8:**
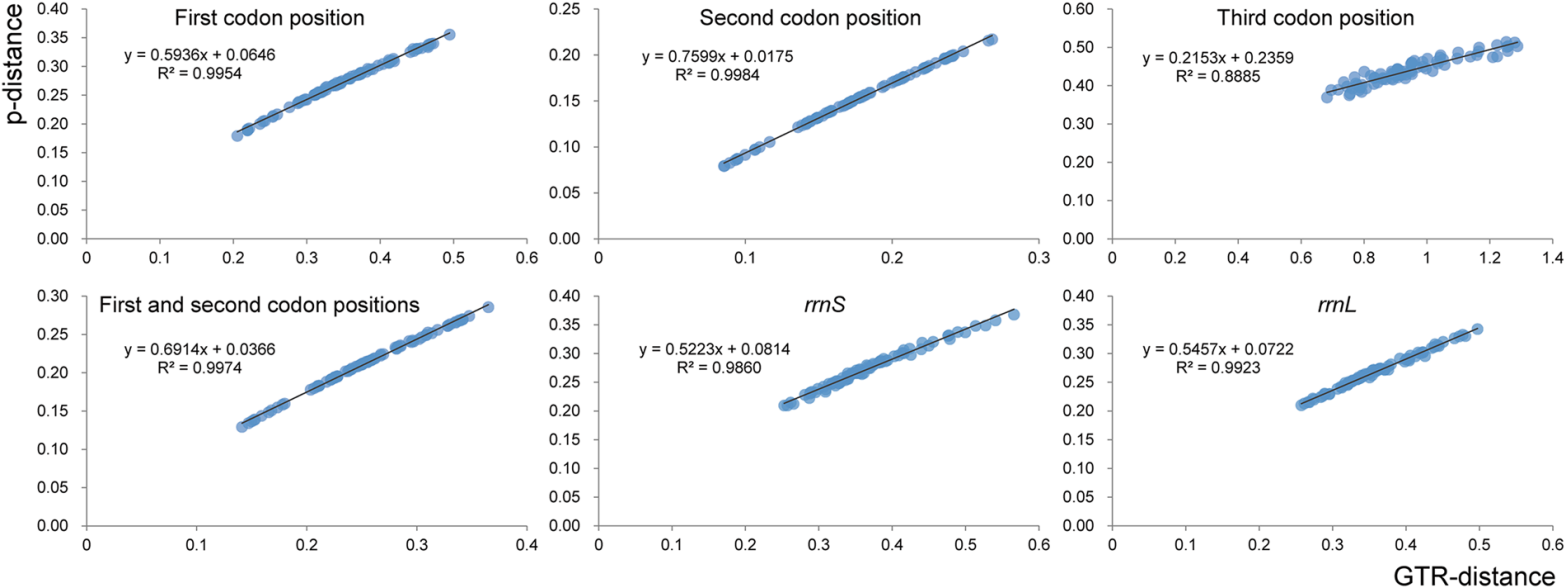
Saturation of the nucleotide substitution tests for 13 protein-coding genes and two rRNA genes (*rrnL* and *rrnS*) of mitochondrial genomes in Membracoidea.

Previously studies confirmed that inclusion or exclusion of third codon positions had a strong influence on phylogenetic reconstruction^51,52^. Our saturation tests on different codon positions of PCGs and two rRNA genes (Fig. 8) showed that third codon positions are more saturated than first and second codon positions, with slopes of 0.2135, 0.5936 and 0.7599, respectively. Nevertheless, in our phylogenetic results, tree topologies were consistent regardless of whether third codon positions were excluded, but excluding third positions slightly increased support for some nodes in ML analyses (BS values: 75 to 84, 84 to 91, 80 to 86 and 87 to 91) (Fig. 8).

## Conclusions

The first comparative analysis of mitochondrial genomes across the superfamily Membracoidea revealed a high level of conservatism in gene order, with all sequenced genes arranged in the putative insect ancestral gene arrangement, but one of the taxa newly sequenced for this study, *J. hyalinus*, had the putative ancestral tRNA cluster *trnW-trnC-trnY* rearranged to *trnY-trnW-trnC*. This is the first reported gene rearrangement in a mitogenome of Membracoidea. Phylogenomic analysis of the concatenated nucleotide sequences of 13 protein-coding genes (PCGs) and two ribosomal RNA genes from all available membracoid mitochondrial genomes supported the monophyly of Membracoidea and paraphyly of Cicadellidae with respect to the treehopper lineage (Aetalionidae + Membracidae). ML and BI analyses yielded topologies that were congruent except for relationships among included representatives of subfamily Deltocephalinae. Exclusion of third codon positions of PCGs improved some node support values in ML analyses. These results suggest that mitochondrial genome sequences are informative of higher level phylogenetic relationships within Membracoidea but may not be sufficient to resolve relationships within some membracoid lineages.

## Electronic supplementary material

Supplementary Information
