# Supplementary material for: Characterization of the complete mitochondrial genomes of Maiestas dorsalis and Japananus hyalinus (Hemiptera: Cicadellidae) and comparison with other Membracoidea
Source: Sci Rep. 2017 Oct 27;7:14197. doi: 10.1038/s41598-017-14703-3 (PMC5660246; doi:10.1038/s41598-017-14703-3)
Supplement: Supplementary file 1 — Supplementary Information [file 41598_2017_14703_MOESM1_ESM.doc]

**Characterization of the complete** **mitochondrial genomes of *Maiestas dorsalis* and *Japananus hyalinus* (Hemiptera: Cicadellidae) and comparison with other Membracoidea**

Yimin Du^1^, Chunni Zhang^1^, Christopher H. Dietrich^2^, Yalin Zhang^1^,Wu Dai^1*^

^1^ Key Laboratory of Plant Protection Resources and Pest Management of Ministry of Education, College of Plant Protection, Northwest A&F University, Yangling, Shaanxi, China

^2^ Illinois Natural History Survey, Prairie Research Institute, University of Illinois at Urbana-Champaign, Illinois, United States of America

*Corresponding Author: [daiwu@nwsuaf.edu.cn](mailto:daiwu@nwsuaf.edu.cn) (WD)

**Supplementary Table S1.** Organization of the *Maiestas dorsalis* mitochondrial genome.

| Name | Start | Stop | Intergenic nucleotide | Length | Start codon | Stop codon | Anticodon | Coding strand |
| --- | --- | --- | --- | --- | --- | --- | --- | --- |
| *trnI* | 1 | 66 | － | 66 |  |  | GAT | ＋ |
| *trnQ* | 64 | 132 | -3 | 69 |  |  | TTG | － |
| *trnM* | 135 | 201 | 2 | 67 |  |  | CAT | ＋ |
| *nad2* | 202 | 1176 | 0 | 975 | ATC | TAA |  | ＋ |
| *trnW* | 1193 | 1261 | 16 | 69 |  |  | TCA | ＋ |
| *trnC* | 1254 | 1316 | -8 | 63 |  |  | GCA | － |
| *trnY* | 1322 | 1388 | 5 | 67 |  |  | GTA | － |
| *cox1* | 1404 | 2957 | 15 | 1554 | ATA | TAA |  | ＋ |
| *trnL2(UUR)* | 2974 | 3038 | 16 | 65 |  |  | TAA | ＋ |
| *cox2* | 3039 | 3720 | 0 | 682 | ATC | T |  | ＋ |
| *trnK* | 3721 | 3792 | 0 | 72 |  |  | CTT | ＋ |
| *trnD* | 3807 | 3876 | 14 | 70 |  |  | GTC | ＋ |
| *atp8* | 3877 | 4029 | 0 | 153 | ATT | TAA |  | ＋ |
| *atp6* | 4023 | 4676 | -7 | 654 | ATG | TAA |  | ＋ |
| *cox3* | 4676 | 5455 | -1 | 780 | ATG | TAA |  | ＋ |
| *trnG* | 5465 | 5527 | 9 | 63 |  |  | TCC | ＋ |
| *nad3* | 5528 | 5881 | 0 | 354 | ATA | TAA |  | ＋ |
| *trnA* | 5883 | 5945 | 1 | 63 |  |  | TGC | ＋ |
| *trnR* | 5946 | 6009 | 0 | 64 |  |  | TCG | ＋ |
| *trnN* | 6009 | 6077 | -1 | 69 |  |  | GTT | ＋ |
| *trnS1(AGN)* | 6076 | 6141 | -2 | 66 |  |  | GCT | ＋ |
| *trnE* | 6145 | 6209 | 3 | 65 |  |  | TTC | ＋ |
| *trnF* | 6208 | 6274 | -2 | 67 |  |  | GAA | － |
| *nad5* | 6274 | 7947 | -1 | 1674 | TTG | TAG |  | － |
| *trnH* | 7948 | 8017 | 0 | 70 |  |  | GTG | － |
| *nad4* | 8017 | 9324 | -1 | 1308 | ATG | TAA |  | － |
| *nad4l* | 9318 | 9593 | -7 | 276 | ATT | TAA |  | － |
| *trnT* | 9596 | 9660 | 2 | 65 |  |  | TGT | ＋ |
| *trnP* | 9661 | 9726 | 0 | 66 |  |  | TGG | － |
| *nad6* | 9729 | 10211 | 2 | 483 | ATT | TAA |  | ＋ |
| *cob* | 10218 | 11354 | 6 | 1137 | ATG | TAA |  | ＋ |
| *trnS2(UCN)* | 11353 | 11417 | -2 | 65 |  |  | TGA | ＋ |
| *nad1* | 11419 | 12350 | 1 | 932 | ATT | T |  | － |
| *trnL1(CUN)* | 12351 | 12416 | 0 | 66 |  |  | TAG | － |
| *rrnL* | 12417 | 13633 | 0 | 1217 |  |  |  | － |
| *trnV* | 13634 | 13699 | 0 | 66 |  |  | TAC | － |
| *rrnS* | 13700 | 14444 | 0 | 745 |  |  |  | － |
| CR | 14445 | 15352 | 0 | 908 |  |  |  | ＋ |

**Supplementary Table S2.** Organization of the *Japananus hyalinus* mitochondrial genome.

| Name | Start | Stop | Intergenic nucleotide | Length | Start codon | Stop codon | Anticodon | Coding strand |
| --- | --- | --- | --- | --- | --- | --- | --- | --- |
| *trnI* | 1 | 67 | － | 67 |  |  | GAT | ＋ |
| *trnQ* | 69 | 137 | 1 | 69 |  |  | TTG | － |
| *trnM* | 145 | 211 | 7 | 67 |  |  | CAT | ＋ |
| *nad2* | 212 | 1189 | 0 | 978 | ATT | TAA |  | ＋ |
| *trnY* | 1209 | 1275 | 19 | 67 |  |  | GTA | － |
| *trnW* | 1349 | 1421 | 73 | 73 |  |  | TCA | ＋ |
| *trnC* | 1414 | 1474 | -8 | 61 |  |  | GCA | － |
| *cox1* | 1487 | 3025 | 12 | 1,539 | ATA | TAA |  | ＋ |
| *trnL2(UUR)* | 3045 | 3110 | 19 | 66 |  |  | TAA | ＋ |
| *cox2* | 3111 | 3789 | 0 | 679 | ATG | T |  | ＋ |
| *trnK* | 3791 | 3860 | 1 | 70 |  |  | CTT | ＋ |
| *trnD* | 3861 | 3934 | 0 | 74 |  |  | GTC | ＋ |
| *atp8* | 3935 | 4093 | 0 | 159 | ATA | TAA |  | ＋ |
| *atp6* | 4087 | 4740 | -7 | 654 | ATG | TAA |  | ＋ |
| *cox3* | 4741 | 5520 | 0 | 780 | ATG | TAA |  | ＋ |
| *trnG* | 5520 | 5582 | -1 | 63 |  |  | TCC | ＋ |
| *nad3* | 5583 | 5936 | 0 | 354 | ATT | TAA |  | ＋ |
| *trnA* | 5957 | 6017 | 20 | 61 |  |  | TGC | ＋ |
| *trnR* | 6017 | 6077 | -1 | 61 |  |  | TCG | ＋ |
| *trnN* | 6077 | 6142 | -1 | 66 |  |  | GTT | ＋ |
| *trnS1(AGN)* | 6142 | 6207 | -1 | 66 |  |  | GCT | ＋ |
| *trnE* | 6208 | 6273 | 0 | 66 |  |  | TTC | ＋ |
| *trnF* | 6276 | 6340 | 2 | 65 |  |  | GAA | － |
| *nad5* | 6342 | 8012 | 1 | 1,671 | TTG | TAA |  | － |
| *trnH* | 8013 | 8075 | 0 | 63 |  |  | GTG | － |
| *nad4* | 8089 | 9396 | 13 | 1,308 | ATG | TAA |  | － |
| *nad4L* | 9390 | 9665 | -7 | 276 | ATT | TAA |  | － |
| *trnT* | 9668 | 9730 | 2 | 63 |  |  | TGT | ＋ |
| *trnP* | 9731 | 9797 | 0 | 67 |  |  | TGG | － |
| *nad6* | 9800 | 10282 | 2 | 483 | ATC | TAA |  | ＋ |
| *cob* | 10287 | 11423 | 4 | 1,137 | ATG | TAA |  | ＋ |
| *trnS2(UCN)* | 11426 | 11490 | 2 | 65 |  |  | TGA | ＋ |
| *nad1* | 11490 | 12425 | -1 | 936 | ATT | TAA |  | － |
| *trnL1(CUN)* | 12426 | 12491 | 0 | 66 |  |  | TAG | － |
| *rrnL* | 12492 | 13699 | 0 | 1,208 |  |  |  | － |
| *trnV* | 13700 | 13764 | 0 | 65 |  |  | TAC | － |
| *rrnS* | 13765 | 14516 | 0 | 752 |  |  |  | － |
| CR | 14517 | 15364 | 0 | 848 |  |  |  | ＋ |

**Figure S1.** AT- and GC-skews in the complete mitochondrial genomes of Membracoidea. Whole, the complete sequences of whole mitogenome; PCGs, protein-coding genes; *rrnL*, large subunit ribosomal RNAs; *rrnS*, small subunit ribosomal RNAs.

**Figure S2.** A + T% of the mitochondrial protein-coding genes in Membracoidea. PCG, protein-coding genes; PCG1, the first codon position of protein-coding genes; PCG2, the second codon position of protein-coding genes; PCG3, the third codon position of protein-coding genes; PCG12, the combined first and second codon positions of protein-coding genes.


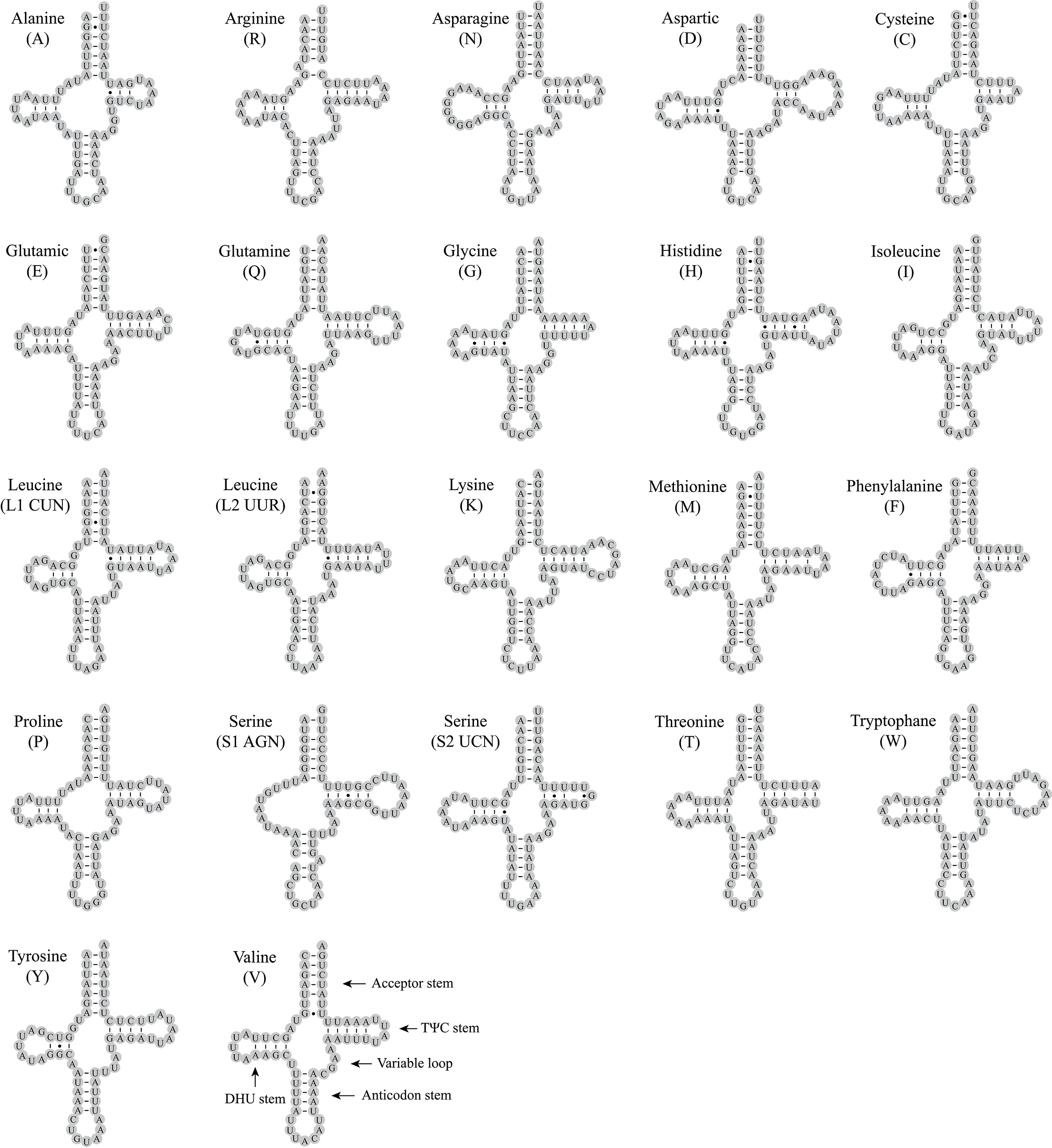


**Figure S3.** Inferred secondary structures of 22 tRNA genes in the mitochondrial genome of *Maiestas dorsalis*. Watson-Crick base pairings are illustrated by lines (-), whereas GU base pairings are illustrated by dots (·).


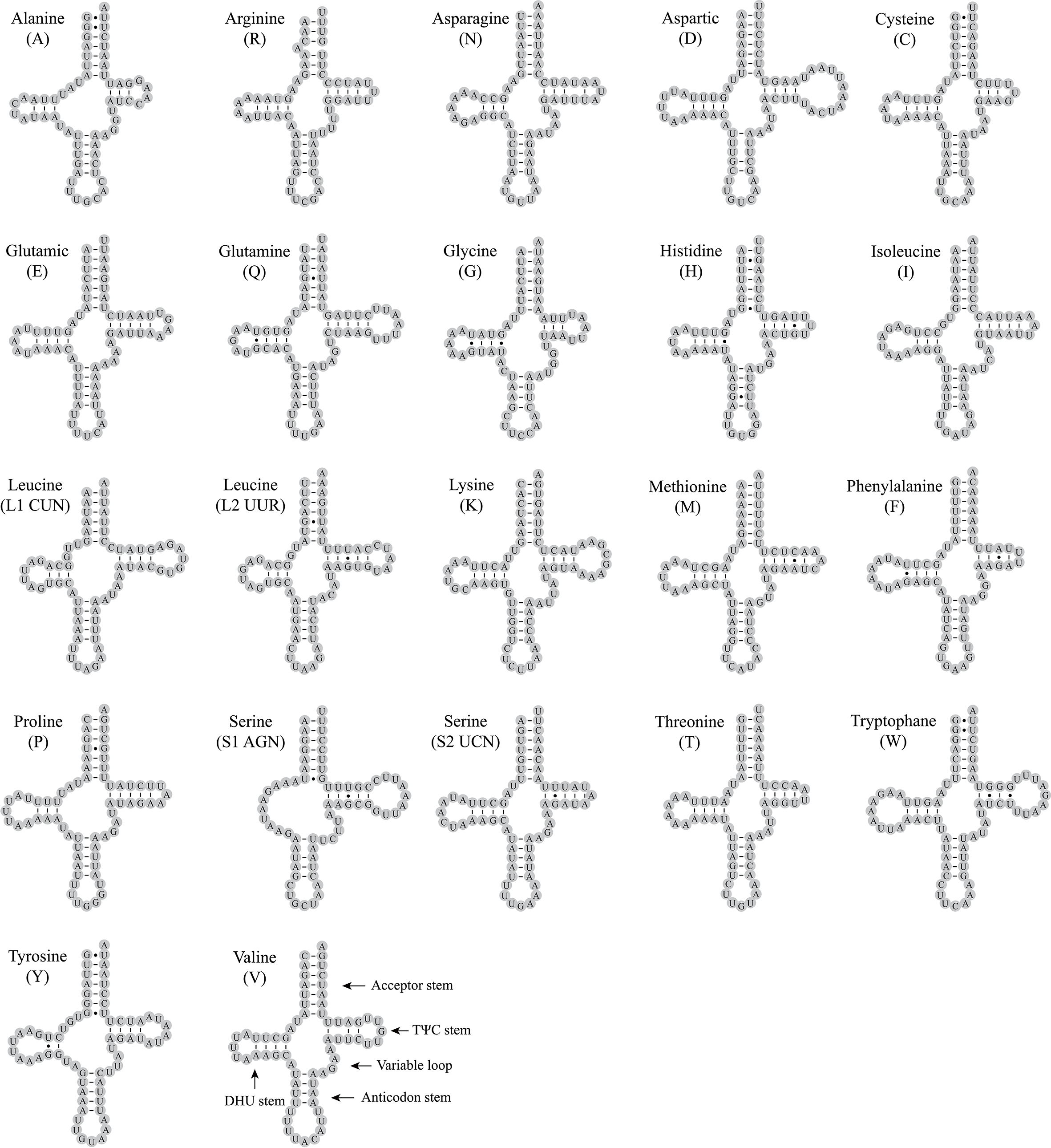


**Figure S4.** Inferred secondary structures of 22 tRNA genes in the mitochondrial genome of *Japananus hyalinus*. Watson-Crick base pairings are illustrated by lines (-), whereas GU base pairings are illustrated by dots (·).

**Supplementary Table S3.** Best partitioning scheme and modelsfor different datasets selected by PartitionFinder.

| Data matrix | Subset Partitions | Models for MrBayes | Models for RAxML | |  |
| --- | --- | --- | --- | --- | --- |
| P123 | P1: (*atp6*_pos1, *cox1*_pos1, *cox2*_pos1, *cox3*_pos1, *cob*_pos1) | GTR+I+G | GTR+I+G | |  |
|  | P2: (*atp6*_pos2, *cox1*_pos2, *cox2*_pos2, *cox3*_pos2, *cob*_pos2) | GTR+I+G | GTR+I+G | |  |
|  | P3: (*atp6*_pos3, *atp8*_pos3, *cox1*_pos3, *cox2*_pos3, *cox3*_pos3, *cob*_pos3, *nad2*_pos3, *nad3*_pos3, *nad6*_pos3) | GTR+I+G | GTR+I+G | |  |
|  | P4: (*atp8*_pos1, *nad1*_pos1, *nad2*_pos1, *nad3*_pos1, *nad4l*_pos1, *nad4*_pos1, *nad5*_pos1, *nad6*_pos1) | GTR+I+G | GTR+I+G | |  |
|  | P5: (*atp8*_pos2, *nad1*_pos2, *nad2*_pos2, *nad3*_pos2, *nad4l*_pos2, *nad4*_pos2, *nad5*_pos2, *nad6*_pos2) | GTR+I+G | GTR+I+G | |  |
|  | P6: (*nad1*_pos3, *nad4l*_pos3, *nad4*_pos3, *nad5*_pos3) | HKY+G | GTR+I+G | |  |
| P12 | P1: (*atp6*_pos1, *cox1*_pos1, *cox2*_pos1, *cox3*_pos1, *cob*_pos1) | GTR+I+G | GTR+I+G | |  |
|  | P2: (*atp6*_pos2, *cox1*_pos2, *cox2*_pos2, *cox3*_pos2, *cob*_pos2) | GTR+I+G | GTR+I+G | |  |
|  | P3: (*atp8*_pos1, *nad1*_pos1, *nad2*_pos1, *nad3*_pos1, *nad4l*_pos1, *nad4*_pos1, *nad5*_pos1, *nad6*_pos1) | GTR+I+G | GTR+I+G | |  |
|  | P4: (*atp8*_pos2, *nad1*_pos2, *nad2*_pos2, *nad3*_pos2, *nad4l*_pos2, *nad4*_pos2, *nad5*_pos2, *nad6*_pos2) | GTR+I+G | GTR+I+G | |  |
| P123R | P1: (12S, 16S, *atp8*_pos1, *nad1*_pos1, *nad2*_pos1, *nad3*_pos1, *nad4l*_pos1, *nad4*_pos1, *nad5*_pos1, *nad6*_pos1) | GTR+I+G | GTR+I+G | |  |
|  | P2: (*atp6*_pos1, *cox1*_pos1, *cox2*_pos1, *cox3*_pos1, *cob*_pos1) | GTR+I+G | GTR+I+G | |  |
|  | P3: (*atp6*_pos2, *cox1*_pos2, *cox2*_pos2, *cox3*_pos2, *cob*_pos2) | GTR+I+G | GTR+I+G | |  |
|  | P4: (*atp6*_pos3, *atp8*_pos3, *cox1*_pos3, *cox2*_pos3, *cox3*_pos3, *cob*_pos3, *nad2*_pos3, *nad3*_pos3, *nad6*_pos3) | GTR+I+G | GTR+I+G | |  |
|  | P5: (*atp8*_pos2, *nad1*_pos2, *nad2*_pos2, *nad3*_pos2, *nad4l*_pos2, *nad4*_pos2, *nad5*_pos2, *nad6*_pos2) | GTR+I+G | GTR+I+G | |  |
|  | P6: (*nad1*_pos3, *nad4l*_pos3, *nad4*_pos3, *nad5*_pos3) | HKY+G | GTR+I+G | |  |
| P12R | P1: (12S, 16S, *atp8*_pos1, *nad1*_pos1, *nad2*_pos1, *nad3*_pos1, *nad4l*_pos1, *nad4*_pos1, *nad5*_pos1, *nad6*_pos1) | GTR+I+G | GTR+I+G | |  |
|  | P2: (*atp6*_pos1, *cox1*_pos1, *cox2*_pos1, *cox3*_pos1, *cob*_pos1) | GTR+I+G | GTR+I+G | |  |
|  | P3: (*atp6*_pos2, *cox1*_pos2, *cox2*_pos2, *cox3*_pos2, *cob*_pos2) | GTR+I+G | GTR+I+G | |  |
|  | P4: (*atp8*_pos2, *nad1*_pos2, *nad2*_pos2, *nad3*_pos2, *nad4l*_pos2, *nad4*_pos2, *nad5*_pos2, *nad6*_pos2) | GTR+G | | GTR+I+G | |
| AA | P1: (*atp6*, *atp8*, *cox2*, *cox3*, *cob*, *nad2*, *nad3*, *nad6*) | MtREV+I+G+F | | MtArt+I+G+F | |
|  | P2: (*cox1*) | MtREV+I+G | | MtArt+I+G | |
|  | P3: (*nad1*, *nad4*, *nad4l*, *nad5*) | MtREV+I+G+F | | MtArt+I+G+F | |
